# Genetic recombination and pathogenicity assessment of porcine reproductive and respiratory syndrome virus 2 strains in China

**DOI:** 10.3389/fvets.2026.1839256

**Published:** 2026-04-30

**Authors:** Yongliang Zhang, Junmei Zhang, Leilei Sun, Qing Li, Wenbo Song, Mingguang Zhou, Gaoyuan Xu, Xibiao Tang, Huanchun Chen, Yunfeng Song

**Affiliations:** 1 State Key Laboratory of Agricultural Microbiology, Huazhong Agricultural University, Wuhan, China; 2 College of Veterinary Medicine, Huazhong Agricultural University, Wuhan, China; 3 College of Informatics, Huazhong Agricultural University, Wuhan, China; 4 Shangdong New Hope & Liuhe Co., Ltd., Qingdao, China; 5 WuHan Golden Draon Group, Wuhan, China; 6 Diagnostic Center Department, Wuhan Keqian Biology Co., Ltd, Wuhan, China

**Keywords:** genetic recombination, molecular epidemiology, NADC30-like, pathogenicity analysis, PRRSV-2, virus isolation

## Abstract

Porcine reproductive and respiratory syndrome virus type 2 (PRRSV-2) has been endemic in China for more than three decades; however, comprehensive epidemiological investigations under field conditions remain limited in recent years. To address this gap, a total of 21,413 clinical samples were collected from 6,974 pig farms across 31 provinces in China between 2023 and 2024 to investigate the epidemiological dynamics of PRRSV-2. Phylogenetic analysis of 1,528 ORF5 sequences identified five major circulating lineages: lineage 1.8 (NADC30-like), lineage 1.5 (NADC34-like), lineage 3.5 (QYYZ-like), lineage 5.1 (VR2332-like), and lineage 8.7 (HP-PRRSV). Among these, lineage 1.8 predominated in both 2023 (47.29%) and 2024 (49.08%). To further characterize the genomic features and pathogenicity of the dominant clinical strains, five recombinant lineage 1.8 PRRSV isolates harboring a characteristic discontinuous 131-amino-acid deletion in nsp2 were successfully isolated. Pathogenicity assessments in piglets revealed distinct differences in virulence among three representative isolates, as reflected by elevated rectal temperatures, reduced average daily weight gain, and high levels of viremia and viral loads in serum, nasal swabs, spleen, lungs, and lymph nodes. Notably, strains with a JXA1-like major parental backbone and an NADC30-like minor parental contribution exhibited higher pathogenicity than those with an NADC30-like major parent and a WUH4-like minor parent. Collectively, this study demonstrates that PRRSV-2 circulation in China is characterized by pronounced seasonality, extensive genetic recombination, and the dominance of genetically diverse lineage 1.8 strains, including recombinants, providing critical insights for targeted surveillance and effective control strategies.

## Introduction

Porcine reproductive and respiratory syndrome (PRRS), caused by porcine reproductive and respiratory syndrome virus (PRRSV), is one of the most economically devastating infectious diseases affecting the global swine industry. The disease is characterized by reproductive failure in sows, respiratory disorders in piglets and growing pigs, and an increased susceptibility to secondary infections (1). PRRSV belongs to the order Nidovirales, family Arteriviridae, and genus Arterivirus. Its genome is a single-stranded, positive-sense RNA of approximately 15 kb, comprising 5′ and 3′ untranslated regions (UTRs) and at least 10 open reading frames (ORFs), including ORF1a, ORF1b, ORF2a, ORF2b, ORF3-5, ORF5a, ORF6, and ORF7 (2). PRRS outbreaks were first reported in Europe and North America in the late 1980s, after which the disease rapidly became endemic in swine-producing regions worldwide (3). According to the International Committee on Taxonomy of Viruses, PRRSV is classified into two distinct species: PRRSV-1 and PRRSV-2, which share approximately 50–70% nucleotide sequence identity (4). PRRSV-1 corresponds to the European genotype and is further divided into four subtypes (5), whereas PRRSV-2 represents the North American genotype and comprises at least nine distinct genetic lineages (6).

Nonstructural protein 2 (nsp2) is the largest and most genetically variable protein encoded by PRRSV, consisting of approximately 980 amino acids. The amino acid sequence identity of nsp2 between PRRSV-1 and PRRSV-2 is only about 40%, highlighting its high degree of divergence. Due to its extensive variability and frequent insertion–deletion events, nsp2 has become an important molecular marker for PRRSV genetic evolution and epidemiological investigations (7). The glycoprotein 5 (GP5), encoded by ORF5, is the major envelope glycoprotein of PRRSV and one of its most variable structural proteins. Consequently, ORF5-based sequence analysis is widely used for phylogenetic classification and molecular epidemiological surveillance of PRRSV (8). In contrast, the membrane (M) protein encoded by ORF6 is among the most conserved structural proteins of PRRSV and is commonly utilized in diagnostic assays due to its high sequence stability and immunogenicity (9).

PRRSV has been present in China for more than 30 years and was first identified in 1995, with early outbreaks reported in Beijing and other coastal regions. To date, PRRSV-2 is the predominant species circulating in mainland China. The period from 2006 to 2008 was marked by the emergence of highly pathogenic PRRSV (HP-PRRSV) variants belonging to lineage 8, which were associated with acute infection, high fever (up to 41 °C), nearly 100% morbidity, and mortality rates of approximately 30%, causing severe economic losses to the swine industry (10). In 2013, a novel PRRSV strain genetically closely related to the NADC30 strain isolated in the United States in 2008 was identified in China and designated as an NADC30-like strain (lineage 1) (11). All NADC30-like strains share a characteristic discontinuous deletion of 131 amino acids in the nsp2 region. Subsequently, the NADC34 strain (IA/2014/NADC34) emerged in the United States in 2016, and NADC34-like strains were first detected in Liaoning Province, China, in 2017 (12). Based on ORF5 sequence variation, NADC30-like strains are classified as lineage 1.5, whereas NADC34-like strains belong to lineage 1.8. In addition, QYYZ-like strains (lineage 3) were initially prevalent in Hong Kong and Taiwan before spreading to mainland China, where representative strains such as QYYZ and GM212 were first isolated in 2010 (13, 14). Currently, lineages 1, 3, 5, and sublineage 8 are co-circulating in mainland China (15).

Previous studies have demonstrated that host immune responses and disease outcomes induced by PRRSV vary substantially among different viral lineages (16–18). The long-term coexistence of multiple PRRSV lineages in China, together with frequent viral mutation and recombination, has greatly complicated epidemiological surveillance and disease control. However, up-to-date nationwide data describing the epidemiological characteristics of PRRSV-2 in mainland China remain limited. In this study, we conducted large-scale epidemiological surveillance of PRRSV-2 in China from 2023 to 2024 by analyzing 21,413 clinical samples collected from 6,974 pig farms across 31 provinces. ORF5 gene sequencing was performed on PRRSV-2–positive samples, yielding 1,528 sequences for phylogenetic analysis. In addition, five PRRSV field strains were isolated, and their genomic characteristics and pathogenicity in piglets were systematically evaluated. This study aims to define the current epidemiological landscape of PRRSV-2 in China, characterize the genetic features of dominant circulating strains, and provide experimental evidence to support the development of effective surveillance and control strategies.

## Results

### Epidemiological characteristics of PRRSV from 2023 to 2024

**Figure 1:**
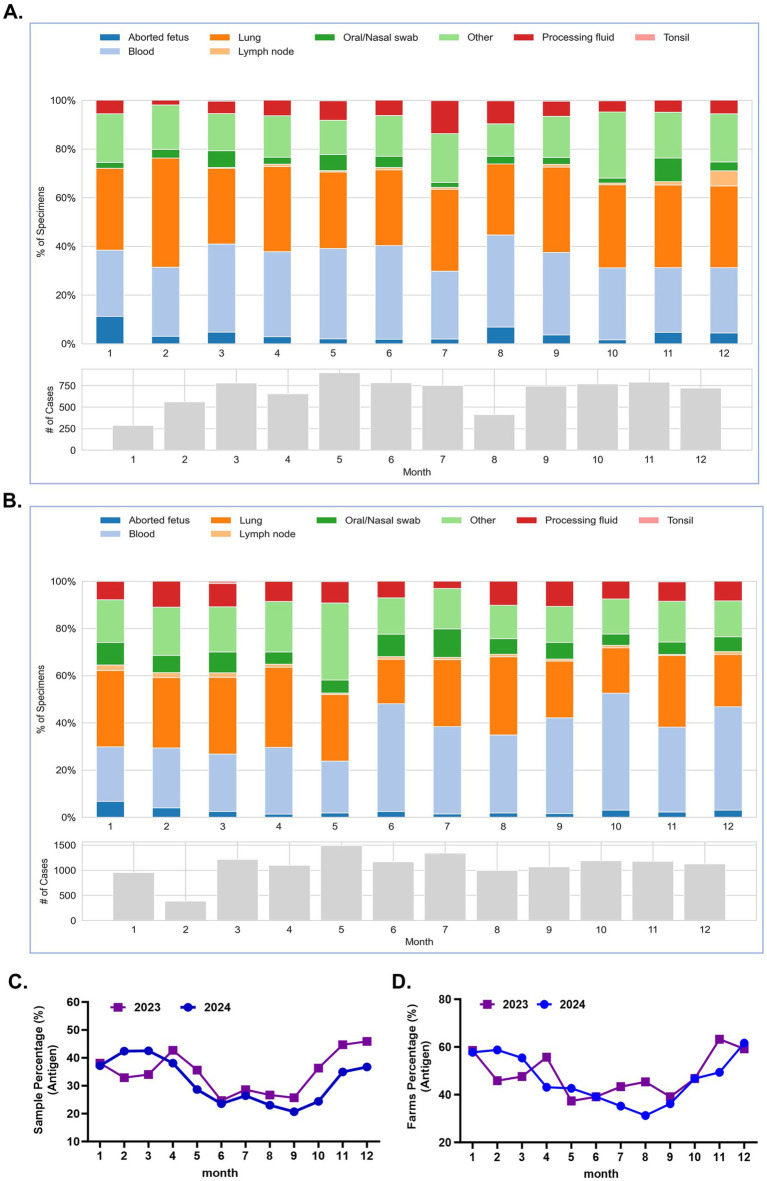
Seasonal epidemiological characteristics of PRRSV. **(A,B)** The percentage distribution of different tissue samples collected each month in 2023 (**A**) and 2024 (**B**). (**C**) Analysis of the positive sample percentages for the 2023 and 2024 based on the detection of the PRRSV ORF6 gene. (**D**) Analysis of the positive farm percentages for the 2023 and 2024 based on the detection of the PRRSV ORF6 gene.

To investigate the epidemiological dynamics of PRRSV-2 in China, a total of 21,413 clinical samples were collected from 6,974 pig farms across 31 provinces between 2023 and 2024. The samples included nasal and oral swabs, tonsils, lymph nodes, blood, lungs, processing fluids, aborted fetuses, and other tissue types (Figures 1A,B). Reverse transcription quantitative polymerase chain reaction (RT-qPCR) detection targeting the PRRSV-2 ORF6 gene, which is highly conserved and exhibits stable expression throughout infection, showed that the farm-level positive rates were 48.82% (1,267/2,595) in 2023 and 46.93% (2,055/4,379) in 2024. At the sample level, the positive rates were 34.73% (2,828/8,142) in 2023 and 30.83% (4,091/13,271) in 2024, indicating that PRRSV-2 infection pressure remained high in mainland China during the study period.

Monthly analysis of PRRSV-2 detection rates in 2023 revealed pronounced temporal variation. The sample-level positive rates ranged from 32.86 to 42.66% between January and May, declined markedly to 24.68–28.57% from June to September, and increased again from October to December, reaching 36.33–45.99% (Figure 1C). A similar pattern was observed at the farm level. The monthly farm-level positive rates ranged from 45.96 to 58.54% between January and May, decreased to 38.11–45.45% during June to September, and subsequently increased to 46.81–63.65% between October and December (Figure 1D). The monthly detection pattern observed in 2024 was largely consistent with that of 2023. Sample-level positive rates ranged from 28.66 to 42.42% between January and May, decreased to 20.69–26.42% from June to October, and increased again to 34.95–36.71% during November and December (Figure 1C). Similarly, farm-level positive rates ranged from 42.68 to 60.26% between January and May, declined to 31.35–39.19% from June to September, and increased to 46.75–61.64% from October to December (Figure 1D). Collectively, analysis of monthly detection rates from 2023 to 2024 demonstrated a clear seasonal pattern of PRRSV-2 prevalence in China, characterized by higher detection rates during winter and spring and lower rates during summer and early autumn.

**Figure 2:**
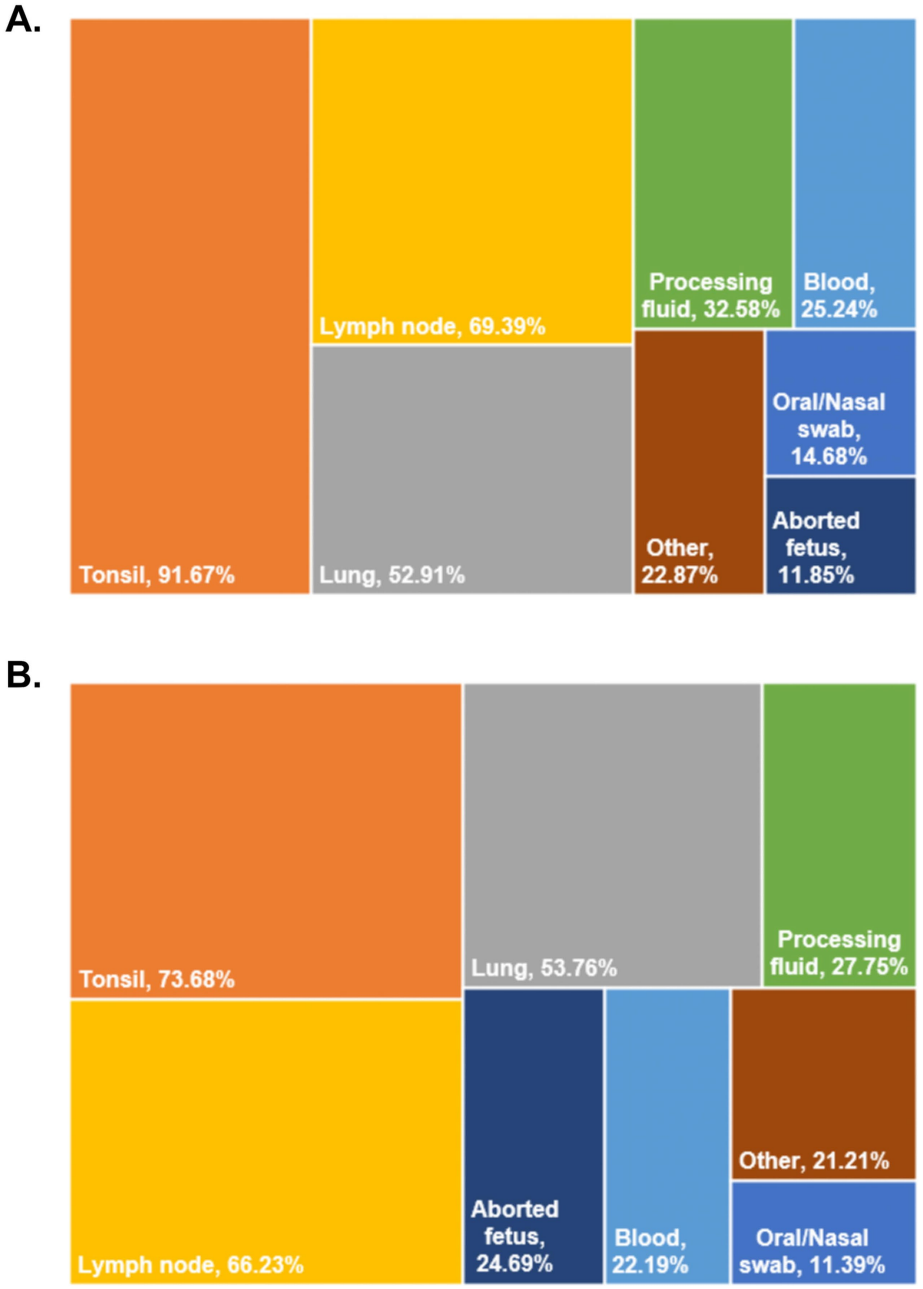
The positive detection rates of different tissue samples in 2023 **(A)** and 2024 **(B)**.

To evaluate PRRSV-2 distribution among different clinical sample types, viral detection rates were analyzed across multiple tissues, including nasal and oral swabs, tonsils, lymph nodes, blood, lungs, processing fluids, and aborted fetuses (Figures 2A,B). In both 2023 and 2024, the highest detection rates were observed in tonsils, lymph nodes, and lung tissues, all exceeding 50%. In contrast, processing fluids showed lower detection rates, at 32.58% in 2023 and 27.75% in 2024. Blood samples exhibited moderate detection rates of 25.24% in 2023 and 22.19% in 2024. The lowest detection rates were observed in nasal and oral swabs and aborted fetuses, with rates of 14.68 and 11.85% in 2023, and 11.39 and 24.69% in 2024, respectively. In general, these results indicates that there is a significant variation in detection rates among different types of clinical samples used for the diagnosis and monitoring of PRRSV-2.

### Genetic evolution analysis of PRRSV-2 strains on pig farms

**Figure 3:**
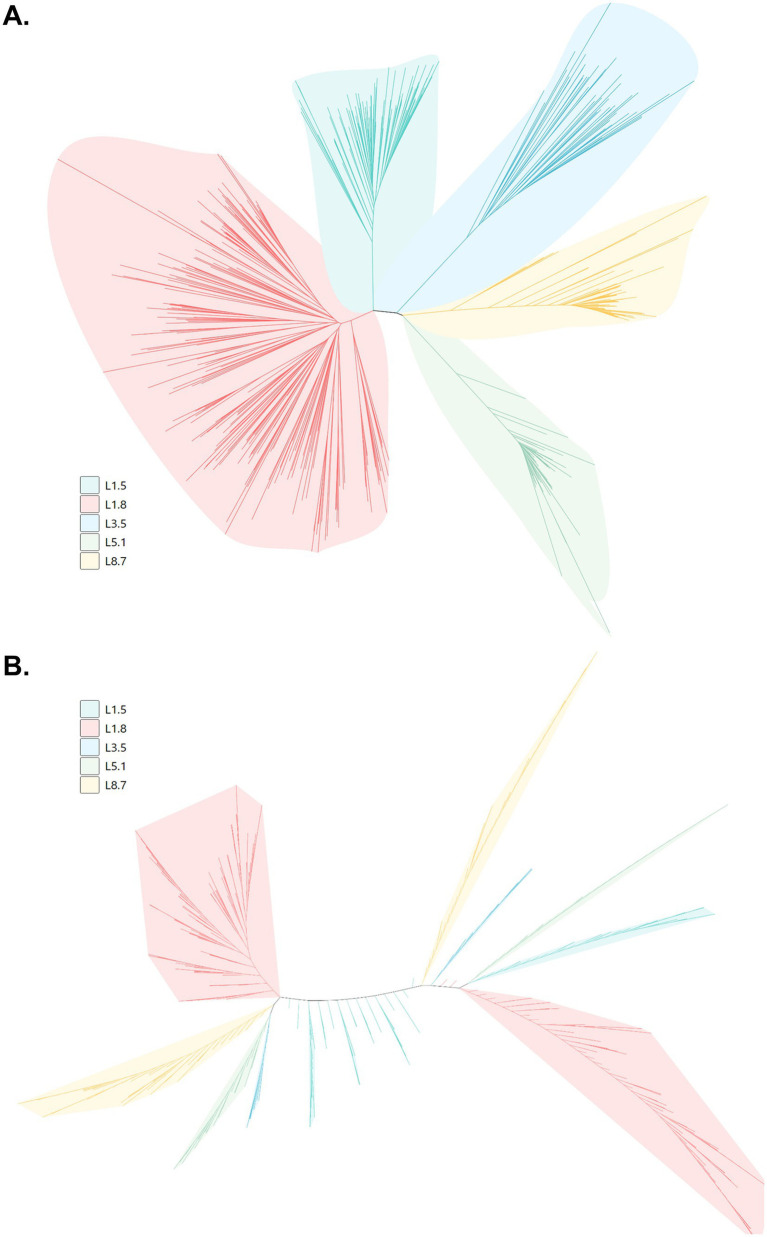
Genetic evolution analysis of PRRSV strains. The phylogenetic trees were constructed based on the 609 PRRSV ORF5 gene sequences obtained in 2023 **(A)** and 919 PRRSV ORF5 gene sequences obtained in 2024 **(B)**.

**Figure 4:**
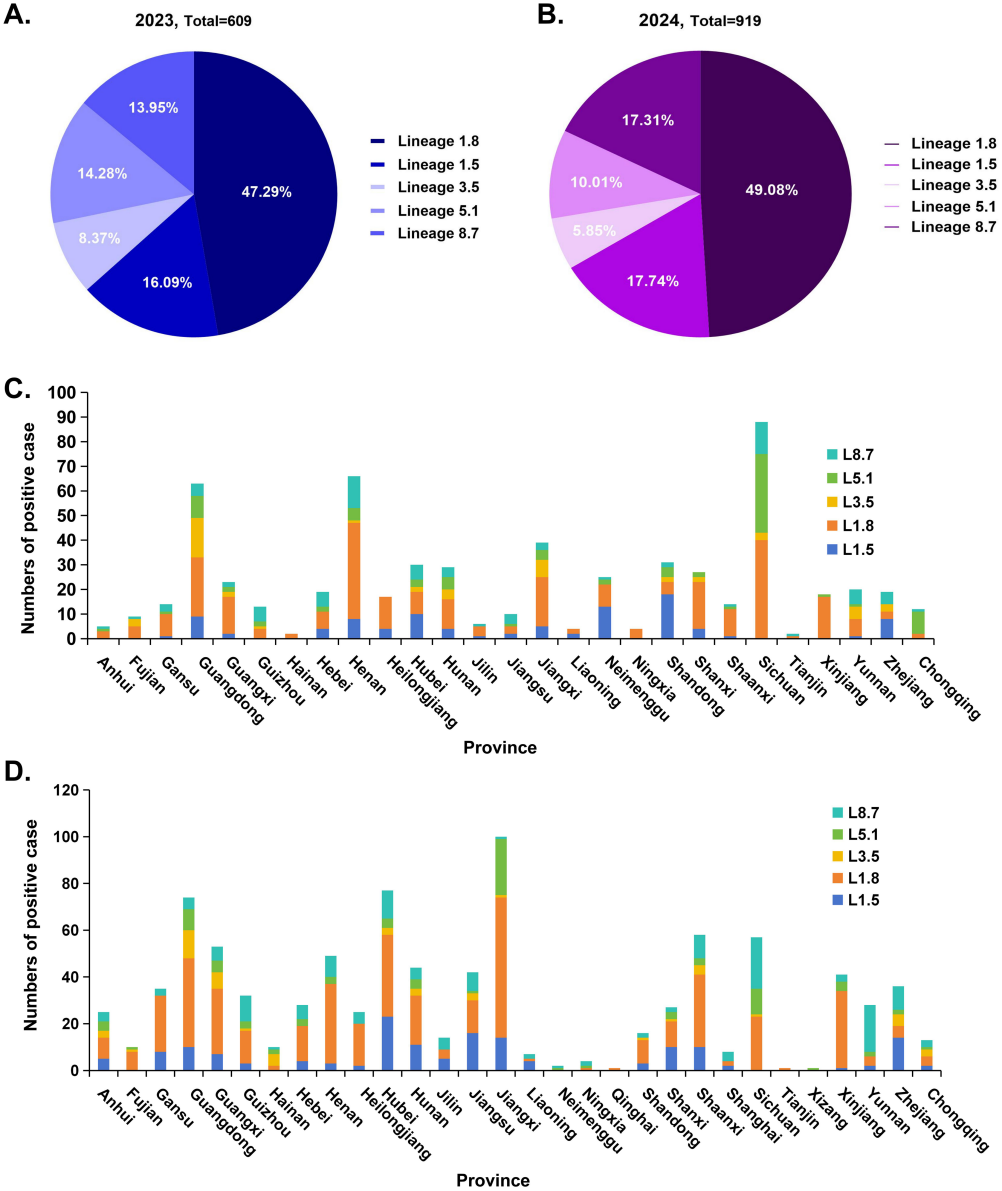
Epidemiological characteristics of various PRRSV lineages from 2023 to 2024. **(A,B)** The positive detection rates of different PRRSV-2 lineages in 2023 **(A)** and 2024 **(B)**. **(C,D)** The number of positive cases of different PRRSV-2 lineages across various provinces in 2023 **(C)** to 2024 **(D)**.

To determine the genetic composition of PRRSV-2 circulating on pig farms in China, ORF5 gene sequencing was performed on PRRSV-2-positive samples. In total, 1,528 ORF5 sequences were obtained, including 609 sequences from 2023 and 919 sequences from 2024. Phylogenetic analysis based on ORF5 sequences revealed that the PRRSV-2 strains clustered into five distinct (sub)lineages: lineage 1.8 (NADC30-like), lineage 1.5 (NADC34-like), lineage 3.5 (QYYZ-like), lineage 5.1 (ATCC-VR2332-like), and lineage 8.7 (HP-PRRSV) (Figures 3A,B). In 2023, lineage 1.8 was the predominant PRRSV-2 sublineage, accounting for 47.29% (288/609) of all sequences, followed by lineage 1.5 (16.09%, 98/609), lineage 8.7 (13.96%, 85/609), lineage 5.1 (14.29%, 87/609), and lineage 3.5 (8.37%, 51/609) (Figure 4A). A similar distribution pattern was observed in 2024. Lineage 1.8 remained dominant, representing 49.08% (451/919) of sequences, followed by lineage 1.5 (17.30%, 159/919), lineage 8.7 (17.74%, 163/919), lineage 5.1 (10.01%, 92/919), and lineage 3.5 (5.88%, 54/919) (Figure 4B). Comparative analysis between 2023 and 2024 showed an increasing proportion of lineage 1.8, lineage 1.5, and lineage 8.7, whereas the relative proportions of lineage 5.1 and lineage 3.5 decreased over the same period (Figures 4A,B). Simultaneously, to further characterize the geographic distribution of PRRSV-2 lineages, the prevalence of different sublineages was compared across provinces. Overall, the lineage distribution patterns in most provinces were consistent with the national trends, with lineage 1.8 being the most prevalent lineage nationwide (Figures 4C,D). Notably, in several provinces, lineage 5.1 also accounted for a relatively high proportion of detected strains, indicating regional variation in lineage composition.

### Isolation, identification, and recombination analysis of PRRSV strains

**Figure 5:**
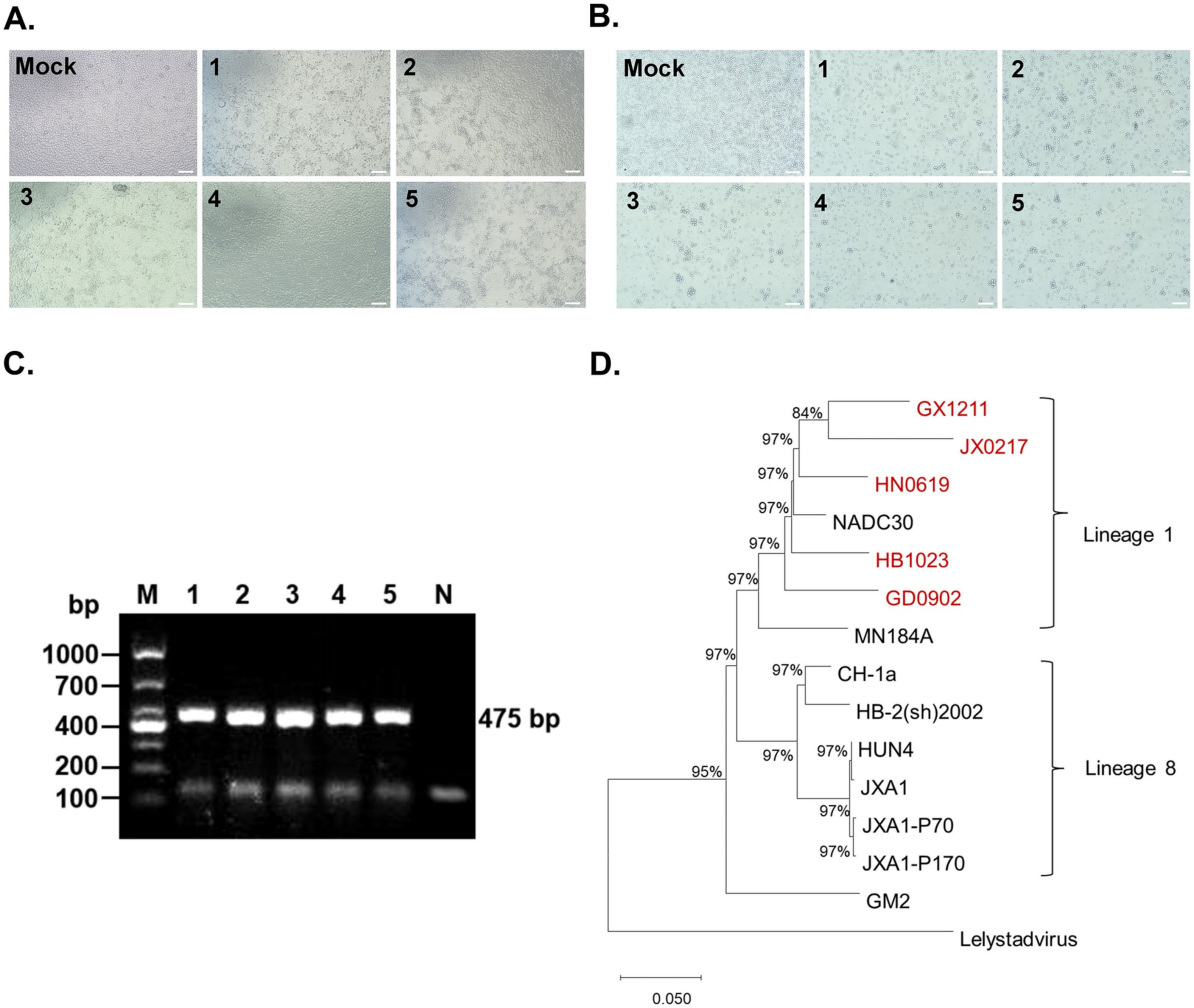
Isolation and identification of PRRSV strains. **(A,B)** Representative images of cytopathic effects (CPE) induced by five PRRSV isolates in Marc-145 cells **(A)** and PAMs **(B)**. Marc-145 cells and PAMs were infected with five PRRSV isolates, respectively, for 24 h at an MOI of 1, followed by the CPE observation under a microscopy. Scale bar: 100 μm. **(C)** Amplification and detection of the partial ORF5 gene sequences from five PRRSV isolates with nucleic acid gel electrophoresis. The numerical labels “1–5” represent the five PRRSV isolated strains: HN0619, HB1203, GD0902, GX1211, and JX0217, respectively. The abbreviations “M” and “N” represent the “marker” and “negative control,” respectively. **(D)** A phylogenetic tree was constructed based on ORF5 gene sequences of the HN0619, HB1203, GD0902, GX1211, JX0217 and reference strains.

Given that lineage 1.8 (NADC30-like) strains were identified as the predominant PRRSV-2 lineage circulating in China, representative field strains were selected for virus isolation and further characterization. Five PRRSV strains, designated HN0619, HB1023, GD0902, GX1211, and JX0217, were successfully isolated from clinical samples collected from PRRSV-positive pig farms located in different regions. Following inoculation of Marc-145 cells, all five isolates induced typical cytopathic effects (CPEs), including cell rounding, shrinkage, aggregation, and subsequent detachment, which became more pronounced as infection progressed (Figure 5A). Similarly, infection of porcine alveolar macrophages (PAMs), the primary target cells of PRRSV *in vivo*, resulted in extensive cellular fragmentation and cell death (Figure 5B). To confirm the identity of the isolated viruses, the PRRSV ORF5 gene was amplified by PCR from infected cell cultures. Agarose gel electrophoresis showed specific amplification products of approximately 475 bps for all five isolates (Figure 5C). Phylogenetic analysis based on the ORF5 gene demonstrated that all five isolates clustered within the NADC30-like group, confirming their classification as lineage 1.8 PRRSV strains (Figure 5D). Taken together, these results indicated the successful isolation of five PRRSV strains.

**Figure 6:**
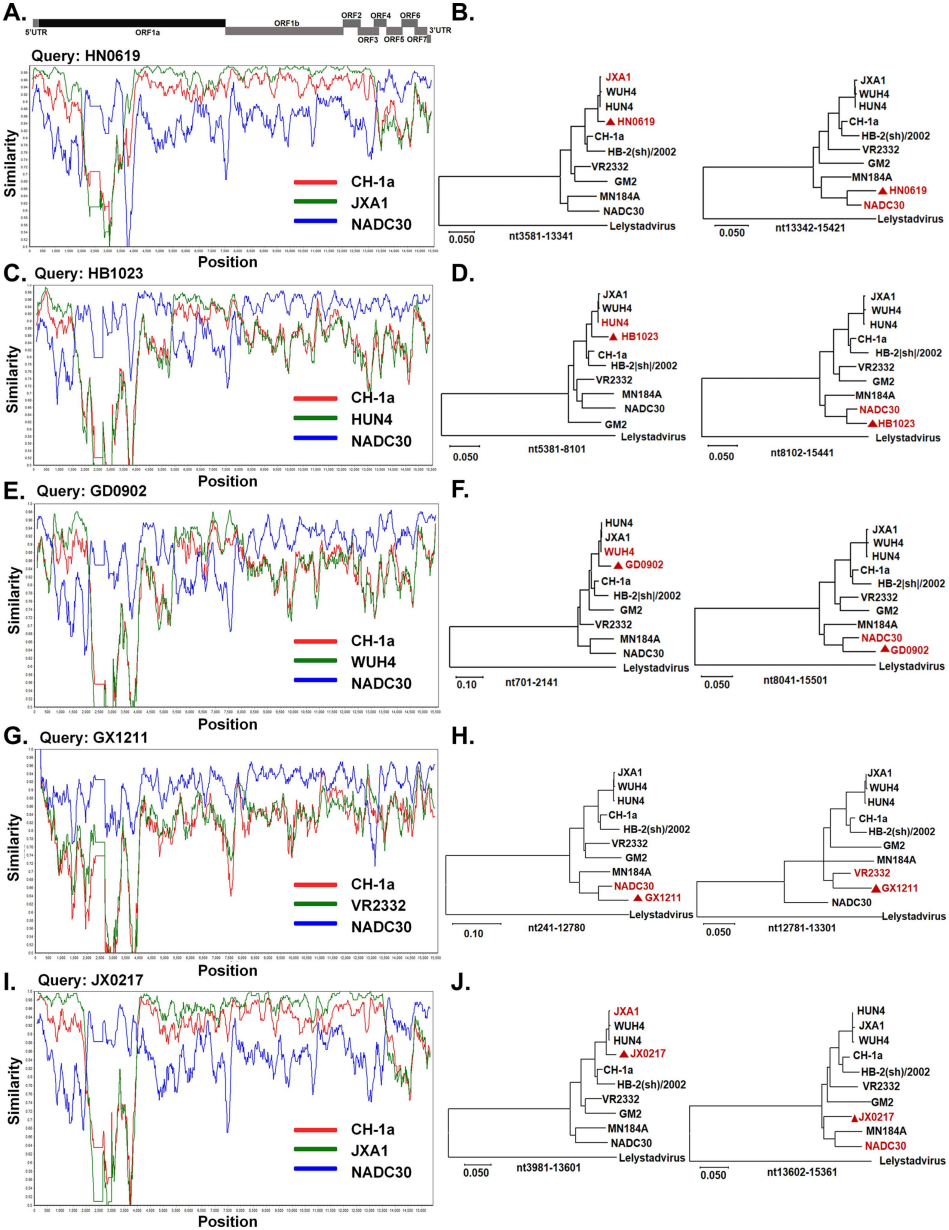
Recombination analysis of PRRSV strains. **(A,C,E,G,I)** Recombination events in PRRSV isolates HN0619 **(A)**, HB1203 (**C**), GD0902 **(E)**, GX1211 **(G)**, and JX0217 **(I)** was conducted by Simplot (version 3.5.1) by comparison with representative reference strains. **(B,D,F,H,J)** Phylogenetic trees analysis was constructed based on different recombination regions of each PRRSV isolates. The target strains HN0619 **(B)**, HB1203 **(D)**, GD0902 **(F)**, GX1211 **(H)**, and JX0217 **(J)** are labeled with red triangle.

Subsequently, to further characterize the genomic features of these isolates, complete genome sequencing was performed. The genome lengths of HN0619, HB1023, GD0902, GX1211, and JX0217 were 15,471 nt, 15,482 nt, 15,575 nt, 15,502 nt, and 15,295 nt, respectively. Recombination analysis revealed that all five PRRSV isolates were recombinant viruses, exhibiting multiple recombination events across the genome. For the HN0619 isolate, three putative recombination breakpoints were identified within the ORF1a and ORF3 regions, delineating four distinct recombinant fragments. Phylogenetic analysis of these regions indicated that a JXA1-like strain served as the major parental strain, whereas NADC30-like strains contributed as minor parental strains (Figures 6A,B). In the HB1023 isolate, three recombination breakpoints were detected within the ORF1a and ORF1b regions, dividing the genome into four recombinant segments. In this case, the NADC30-like strain acted as the major parental strain, with a HUN4-like strain serving as the minor parental strain (Figures 6C,D). The GD0902 isolate contained four recombination breakpoints located within ORF1a and ORF1b, resulting in five recombinant regions. Phylogenetic analysis indicated that the NADC30-like strain was the major parental strain, whereas a WUH4-like strain functioned as the minor parental strain (Figures 6E,F). For the GX1211 isolate, two recombination breakpoints were identified within the ORF3 region, dividing the genome into three recombinant fragments. In this isolate, the NADC30-like strain was identified as the major parental strain, with a VR2332-like strain serving as the minor parental strain (Figures 6G,H). In the JX0217 isolate, three recombination breakpoints were detected within ORF1a and ORF4, generating four recombinant regions. Phylogenetic analysis demonstrated that a JXA1-like strain served as the major parental strain, whereas NADC30-like strains contributed as minor parental strains (Figures 6I,J).

**Figure 7:**
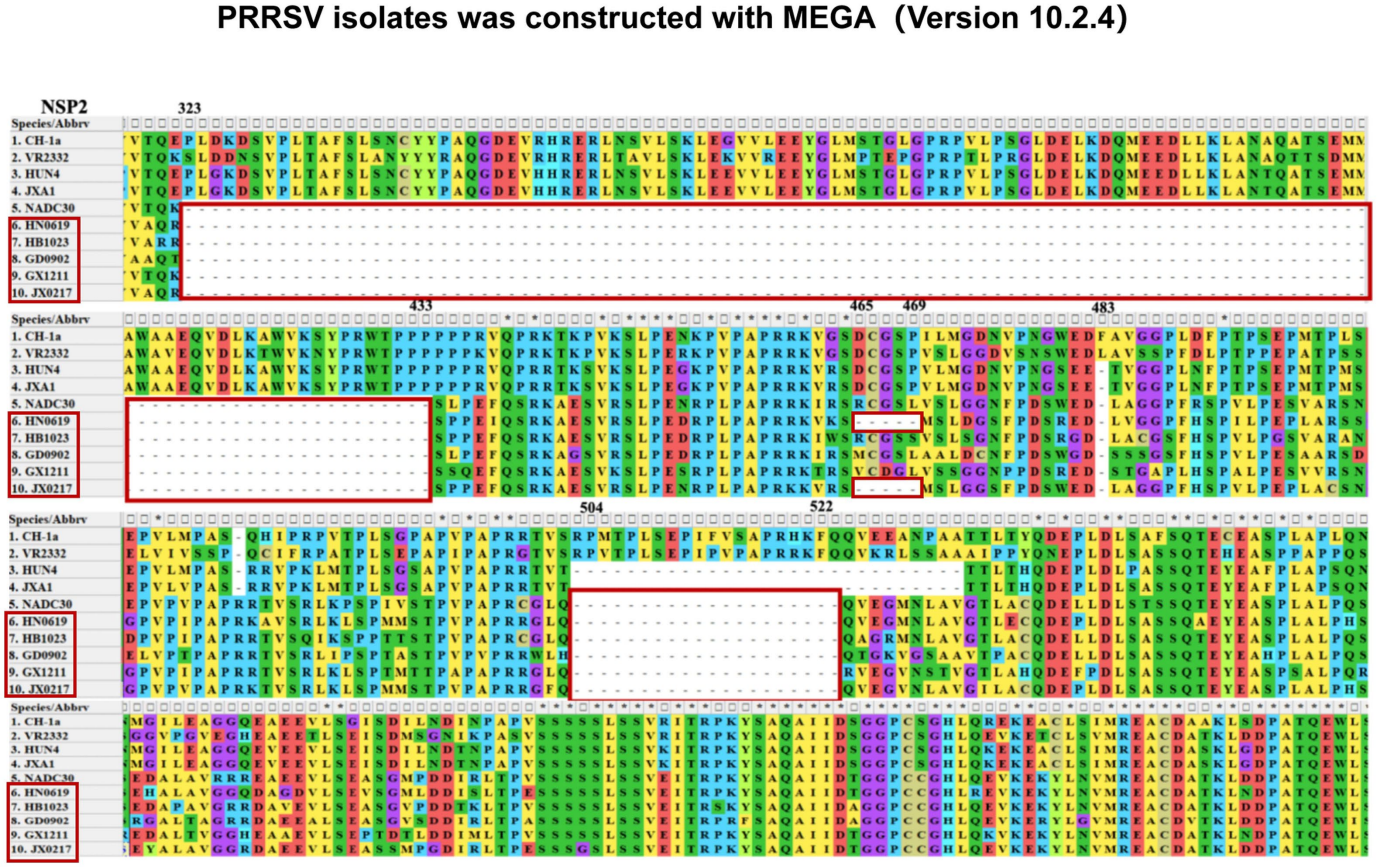
Alignment of the nsp2 amino acid sequences of the five PRRSV isolates was constructed with MEGA (version 10.2.4). The deleted amino acid regions are indicated with red boxes.

Given that nsp2 is the most variable nonstructural protein of PRRSV and frequently undergoes amino acid deletions, the nsp2 sequences of the five isolates were compared with those of representative reference strains, including CH-1a, VR2332, HUN4, and JXA1. All five isolates exhibited a characteristic discontinuous deletion of 131 amino acids in nsp2, consisting of deletions at amino acid positions 323–433, 483, and 504–522, which is consistent with the deletion pattern of NADC30-like strains. Notably, the HN0619 and JX0217 isolates contained an additional deletion of five amino acids at positions 465–469 (Figure 7). Taken together, these results demonstrate that the NADC30-like PRRSV strains currently circulating in China exhibit extensive genetic recombination, involving multiple parental lineages and diverse recombination patterns, highlighting the increasing genetic complexity of PRRSV-2 field strains.

### Pathogenicity assessment of PRRSV isolates in piglets

**Figure 8:**
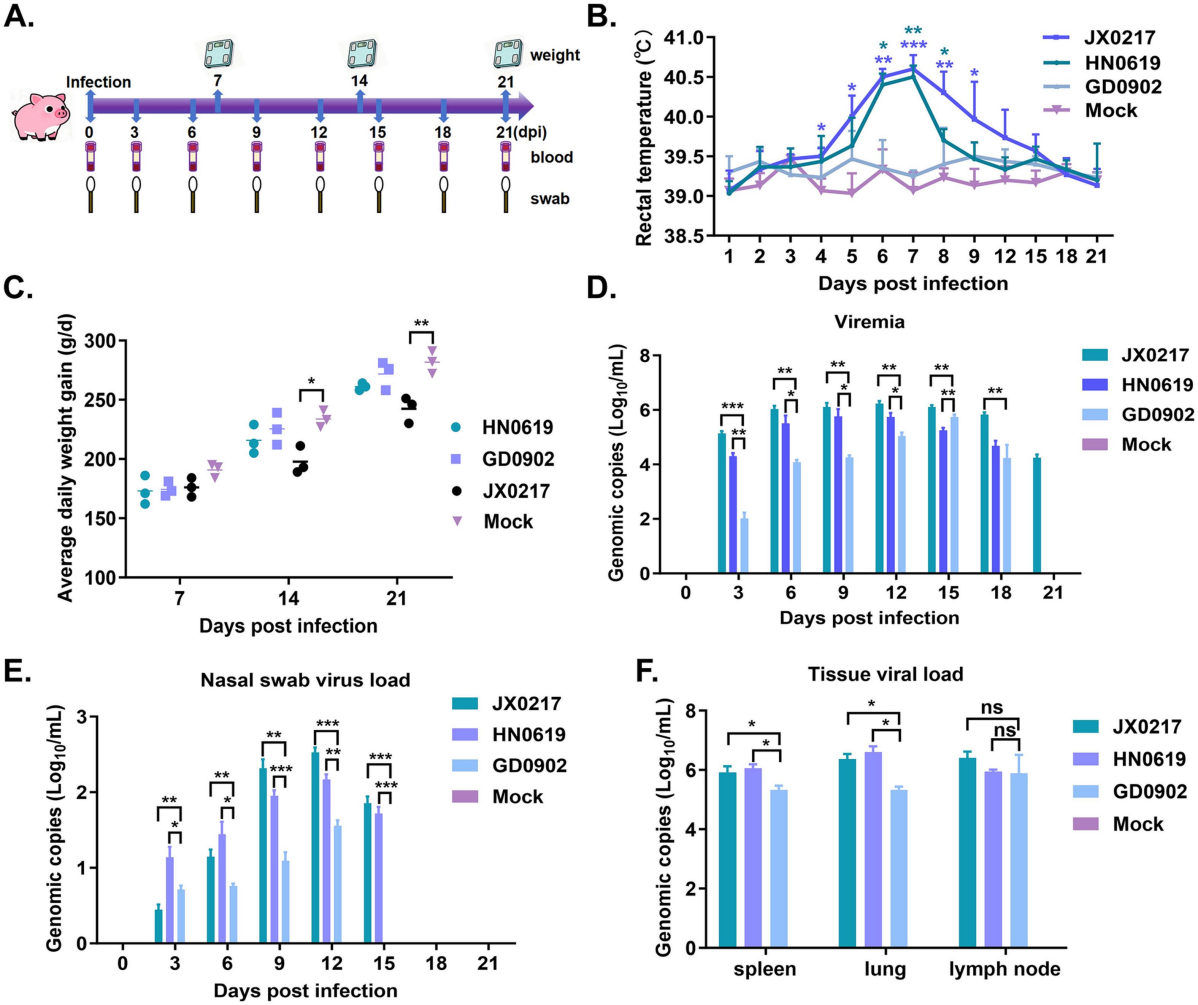
Pathogenicity assessment of PRRSV isolates in piglets. **(A)** The schematic diagram of the animal trial. **(B–E)** The rectal temperature **(B)**, average daily weight gain **(C)**, viremia kinetics **(D)**, and nasal swab viral load kinetics **(E)** were monitored after PRRSV isolates-challenged. **(F)** Detection of the viral load in tissues 21 days post-PRRSV infection. Statistical significance was determined at *p-*values < 0.05, indicated as *p < 0.05, **0.001 < *p* < 0.01, ***p < 0.001; ns, indicated no statistical difference.

The infectious titers of the five PRRSV isolates were determined prior to animal challenge. The 50% of the tissue culture infection dose (TCID_50_) values of isolates JX0217, HN0619, and GD0902 were 10^6.65^, 10^6.25^, and 10^6.38^ TCID_50_/mL, respectively, which were higher than those of HB1023 (10^5.21^ TCID_50_/mL) and GX1211 (10^5.14^ TCID_50_/mL). Based on viral titers, three isolates (JX0217, HN0619, and GD0902) were selected for subsequent pathogenicity evaluation in piglets. Twenty-eight-day-old piglets were intranasally inoculated with each selected isolate at a dose of 2 × 10^6^ TCID_50_, and clinical parameters including rectal temperature, body weight, viremia, viral shedding, and tissue viral loads were monitored over a 21-day period (Figure 8A).

Piglets infected with the JX0217 and HN0619 isolates exhibited a significant increase in rectal temperature beginning at 5 days post-infection (dpi), which persisted until 9 dpi. In contrast, no marked elevation in body temperature was observed in piglets infected with the GD0902 isolate throughout the monitoring period (Figure 8B). Body weight monitoring revealed that piglets infected with JX0217 and HN0619 showed significantly reduced weight gain compared with the mock-infected group. In contrast, piglets infected with GD0902 exhibited only a slight and statistically non-significant reduction in average daily weight gain (Figure 8C). Viremia was detected in all infected groups as early as 3 dpi and persisted until 18 dpi. Notably, viremia in the JX0217-infected group remained detectable through 21 dpi, the final day of observation. During the early stage of infection (3–6 dpi), viral RNA levels in the sera of the JX0217- and HN0619-infected piglets were markedly higher than those observed in the GD0902-infected group (Figure 8D). Analysis of nasal swabs demonstrated that viral shedding via the respiratory tract commenced at 3 dpi and continued until 15 dpi, with no detectable viral RNA at 21 dpi. Piglets infected with JX0217 and HN0619 exhibited higher levels of viral shedding compared with those infected with GD0902, indicating more robust viral replication in the upper respiratory tract (Figure 8E). At 21 dpi, all piglets were euthanized to assess viral distribution in different tissues. Viral RNA was detected in the spleen, lungs, and hilar lymph nodes of all infected groups. Piglets infected with JX0217 and HN0619 exhibited slightly higher viral loads in these tissues compared with those infected with GD0902 (Figure 8F). In summary, these results demonstrate distinct differences in pathogenicity among the three PRRSV-2 isolates. JX0217 and HN0619 exhibited higher virulence, as reflected by increased body temperature, reduced weight gain, prolonged viremia, enhanced viral shedding, and higher tissue viral loads, whereas GD0902 showed comparatively lower pathogenicity.

## Discussion

PRRSV continues to pose a substantial threat to the global swine industry, largely due to its extensive genetic diversity, frequent recombination, and persistent circulation in pig populations (19). The predominant strain circulating in China is PRRSV-2, encompassing lineages 1, 3, 5, 8, and 9 (20, 21). Systematic epidemiological surveillance of PRRSV-2 has provided critical insights for clinical prevention and control efforts (22, 23). However, over the past 2 years, there has been a gap in comprehensive monitoring of PRRSV-2 within China. In this study, we conducted nationwide epidemiological surveillance of PRRSV-2 in China from 2023 to 2024 by integrating large-scale field sampling, ORF5-based phylogenetic analysis, whole-genome sequencing, and *in vivo* pathogenicity assessment. Our results demonstrate that PRRSV-2 remains widely prevalent in mainland China, exhibits a pronounced seasonal pattern, and is dominated by genetically complex recombinant strains belonging to lineage 1.8. Furthermore, pathogenicity experiments revealed marked differences in virulence among representative field isolates, which were closely associated with distinct recombination patterns.

The nationwide surveillance data revealed that PRRSV-2 infection pressure in China remained consistently high throughout 2023 and 2024, with nearly half of the monitored pig farms testing positive. Importantly, a clear seasonal pattern was observed, characterized by increased detection rates during winter and spring and reduced prevalence during summer and early autumn. This seasonal trend is highly consistent with epidemiological patterns reported in other major swine-producing countries, including the United States (24), suggesting that climatic conditions and seasonal management practices may play a conserved role in shaping PRRSV transmission dynamics. From a practical perspective, these findings emphasize the importance of strengthening PRRSV surveillance, vaccination programs, and biosecurity measures during periods of elevated risk, particularly from late autumn to early spring.

Research indicates that, depending on the stage of infection, the distribution of PRRSV in swine exhibits distinct temporal and spatial dynamics, as well as tissue tropism variations. This variability renders the selection of tissue sampling sites a critical factor influencing the detection of PRRSV antigens, antibodies, and viral isolation (25). During the acute phase of infection, viral load in the bloodstream is elevated, and viral shedding occurs via the respiratory tract; consequently, blood samples and tonsillar swabs are considered the preferred specimens (26, 27). Conversely, in the persistent infection phase, the concentration of viral particles in the blood declines sharply, while the virus continues to reside in lymphoid tissues. At this stage, samples from bronchial lymph nodes, mandibular lymph nodes, inguinal lymph nodes, and lung tissue yield more reliable detection results (28). However, some studies have demonstrated that, due to the convenience of sample collection, tongue tip fluid, processing fluid, and blood can also play a crucial role in the monitoring of PRRSV (29, 30). In this study, analysis of PRRSV-2 detection rates across different clinical sample types revealed pronounced tissue-dependent differences. Tonsils, lymph nodes, and lung tissues consistently showed higher viral detection rates than blood samples, nasal and oral swabs, and processing fluids. These findings are in line with previous studies demonstrating that PRRSV establishes persistent infection in lymphoid tissues, even after viremia declines during later stages of infection (31). Although blood and processing fluid samples are convenient for routine surveillance, our results highlight the importance of incorporating lymphoid and respiratory tissues into diagnostic and monitoring strategies, particularly when assessing persistent or subclinical infections. A combined, multi-sample approach may therefore provide a more accurate representation of PRRSV circulation at the herd level.

GP5, a glycosylated envelope protein encoded by the ORF5 gene, is a major structural protein and immunoprotective antigen of PRRSV, and it represents one of the most significantly variable structural proteins of the virus. Therefore, phylogenetic analysis based on ORF5 sequences is essential for understanding the evolutionary dynamics of PRRSV (32). ORF5-based phylogenetic analysis revealed that multiple PRRSV-2 lineages are currently co-circulating in mainland China, with lineage 1.8 (NADC30-like) being the predominant lineage during both 2023 and 2024. In addition to lineage 1.8, lineages 1.5, 8.7, 5.1, and 3.5 were also detected at varying frequencies, underscoring the highly heterogeneous genetic landscape of PRRSV-2 in China. Notably, comparative analysis between the 2 years indicated an increasing proportion of lineage 1.8, lineage 1.5, and lineage 8.7 strains. The continued expansion of these lineages may reflect their enhanced adaptability to current immune environments shaped by vaccination and prior exposure, although further studies are required to clarify the underlying driving forces. Based on these epidemiological findings, we recommend that large-scale pig farms in China prioritize monitoring and controlling lineages 1.8 and 1.5. Additionally, research focused on vaccines targeting these two lineages holds significant clinical relevance for the effective prevention and control of PRRSV in the field.

Five NADC30-like isolates (HN0619, HB1023, GD0902, GX1211, and JX0217) were collected from various pig farms across China. Whole-genome sequencing and recombination analysis demonstrated that all five NADC30-like field isolates characterized in this study were recombinant viruses, involving multiple parental lineages such as JXA1-like, NADC30-like, WUH4-like, HUN4-like, and VR2332-like strains. These findings provide further evidence that recombination has become a dominant evolutionary mechanism driving PRRSV-2 diversification in China. The extensive recombination observed across different genomic regions highlights the increasing genetic complexity of circulating PRRSV strains and poses significant challenges for molecular surveillance, vaccine efficacy, and disease control. In particular, the involvement of vaccine-related or historically prevalent strains as parental backbones raises concerns regarding the long-term evolutionary consequences of widespread vaccination under field conditions. Importantly, pathogenicity assessment in piglets revealed a clear association between recombination patterns and virulence. Isolates JX0217 and HN0619, which possessed a JXA1-like strain as the major parental backbone and an NADC30-like strain as the minor parent, exhibited higher pathogenicity than GD0902, which harbored an NADC30-like major parent and a WUH4-like minor parent. These differences were consistently reflected across multiple clinical and virological parameters, including body temperature, weight gain, viremia, viral shedding, and tissue viral loads. Although the molecular mechanisms underlying these differences remain to be elucidated, our findings suggest that the genetic composition of recombinant PRRSV strains, particularly the identity of the major parental backbone, may play a critical role in shaping viral virulence. Interestingly, we identified an additional deletion of five amino acids (aa. 465–469) in the nsp2 protein of the HN0619 and JX0217 strains, which exhibit higher pathogenicity. We hypothesize that this deletion may be associated with the enhanced virulence of these strains, warranting further in-depth investigation. This observation underscores the necessity of integrating genomic recombination analysis with functional pathogenicity assessment when evaluating the potential risk posed by emerging PRRSV variants. Finally, we acknowledge that the sample size used in this study (*n* = 3 per group) is relatively small. PRRSV infection is known to exhibit considerable inter-individual variability in terms of viremia, clinical signs, and host immune responses. Therefore, the virulence differences observed in this study should be interpreted with caution. Future studies with larger cohort sizes are necessary to validate the statistical significance and biological reproducibility of the observed differences.

## Conclusion

This study provides a comprehensive overview of the current epidemiological landscape, genetic diversity, and pathogenic characteristics of PRRSV-2 in mainland China from 2023 to 2024. The dominance of recombinant lineage 1.8 strains, together with their marked variability in pathogenicity, highlights the dynamic and evolving nature of PRRSV under field conditions. These findings provide important implications for PRRSV surveillance, vaccine development, and disease control strategies. Continuous nationwide monitoring that integrates molecular epidemiology with functional characterization of field isolates will be essential for anticipating the emergence of high-risk variants and mitigating the long-term impact of PRRS on the swine industry.

## Materials and methods

### Samples collection

In 2023–2024, 21,413 porcine tissue samples from 6,974 pig farms across 31 provinces between 2023 and 2024 were collected, including nasal and oral swab, tonsil, lymph node, blood, lung, processing fluid, aborted fetus and other tissue samples. After three freeze–thaw cycles, the samples were stored at −80 °C for subsequent detection and analysis.

### RT-qPCR

Approximately 5.0 g or 300 μL of each sample was homogenized separately in phosphate-buffered saline (PBS). According to the manufacturer’s instructions, viral RNA was extracted using the viral RNA extraction kit (Takara, China) and then the TIANScriptII RT Kit (TIANGEN, China) was used to acquire cDNA through reverse transcription. Then, the viral load from tissues samples was detected using cDNA template, employing specific primers and a probe (Table 1). A standard curve was established through tenfold serial dilutions of a plasmid containing the PRRSV ORF6 fragment. Cycle threshold values obtained from RT-qPCR were applied to this standard curve to calculate viral genome copy numbers in each sample.

**Table 1:** The primers and probe used in this study.

Primers and probe	Sequences (5′–3′)
ORF6-F	TTGCTAGGCCGCAAGTAC
ORF6-R	ACGCCGGACGACAAATGC
ORF6-probe	FAM-CTGGCCCCTGCCCACCAC-BHQ1

### ORF5 gene sequencing and genetic evolution analysis

The ORF5 gene sequences of PRRSV-2 positive samples were amplified using the obtained cDNA template and sequenced by PCR using the primers listed in Table 2, resulting in a total of 1,528 gene sequences. Subsequently, phylogenetic and molecular evolutionary analyses were conducted using MEGA version X (33). A neighbor-joining phylogenetic tree was generated in MEGAX under the Poisson model, utilizing the multiple sequence alignment as input. Bootstrap support was assessed with 1,000 replicates, while other parameters remained at their defaults. The resulting neighbor-joining tree was visualized and annotated using iTol (34). The reference PRRSV strain sequences were presented in Table 3.

**Table 2:** The primers used in this study.

Primers	Sequences (5′–3′)	Products
ORF5-F	GAGGTGGGCAACYGTTTTAG	475 bps
ORF5-R	CAMGMGTAGCGCCAGGACA	

**Table 3:** The sequences of reference PRRSV strains.

Strains	Country	Accession numbers
CH-1a	China	AY032626
JXA1	China	EF112445
HuN4	China	EF635006
HB-2(sh)/2002	China	AY262352.1
GM2	China	JN662424
WUH4	China	JQ326271.1
VR2332	America	EF536003.1
NADC30	America	JN654459
MN184A	America	DQ176019
Lelystad virus	Netherlands	M96262
JXA1-P70	China	FJ548852.1
JXA1-P170	China	JQ804986.1

### Virus isolation and identification

The positive tissues were filtered through 0.22 μm filters and inoculated onto PAMs, which were isolated from 28-day-old specific pathogen-free (SPF) piglets and cultured in Roswell Park Memorial Institute (RPIM) 1,640 medium (Gibco, NY, USA) supplemented with 10% FBS (Gibco, NY, USA) and penicillin–streptomycin at 37 °C in a humidified atmosphere of 5% CO_2_. When the virus infected PAMs exhibited CPE, the virus was harvested through three freeze–thaw cycles. The PRRSV isolates were purified using the plaque assay in marc-145 cells and identified by PCR targeting ORF5 gene sequence. The primers were listed in the followed Table 2.

### Genome sequencing and recombination analysis

PRRSV isolates (HN0619, HB1023, GD0902, GX1211, and JX0217) was sequentially passaged on PAMs, and the genomic RNA of third passage strains were extracted from the infected PAMs for whole-genome sequencing by Tanpu Biotechnology Co., Ltd. (Shanghai, China). The phylogenetic tree was constructed using the neighbor-joining method with 1,000 bootstrap replicates in MEGA-X to evaluate genetic evolution. Recombination analysis was conducted by Simplot (version 3.5.1) with a window width of 200 base pairs (bps) and a step size of 20 bps to identify potential parental strains (35).

### Pathogenicity in piglets

Crossbred healthy SPF piglets (Landrace ×Large White, 28-day-old, male, 9 ~ 11 kg) were purchased and housed in SPF barrier facilities at the Huazhong Agriculture University (Wuhan, China). All piglets were maintained at an ambient temperature of 20–25 °C in an environmentally controlled room by air conditioning and illumination (12 h light and dark cycles). Each cage was equipped with a feeder and water nipple to allow free access to food and drinking water.

Twelve piglets were randomly divided into four groups: JX0217 (*n* = 3), HN0619 (*n* = 3), GD0902 (*n* = 3) and a mock group (*n* = 3). Piglets in the infection groups were inoculated intranasally with viral strains (2 × 10^6^ TCID_50_, 2 mL). The control group received an equivalent volume of DMEM medium via the same administration route. Following inoculation, rectal temperatures and body weights were measured at 0, 3, 6, 9, 12, 15, 18, and 21 dpi. Samples of serum and nasal swabs were collected at 0, 3, 6, 9, 12, 15, 18, and 21 dpi for RT-qPCR analyses. At 21 dpi, all piglets were euthanized for necropsy, and tissue samples were collected for viral load detection using RT-qPCR.

### Statistical analysis

Statistical analyses were constructed with GraphPad Prism 10.1.2 (GraphPad Software Version 10.1.2), with data presented as mean ± standard deviation (SD). Comparisons were performed through one-way or two-way ANOVA followed by Tukey’s test. Statistical significance was determined at *p* values < 0.05, indicated as *p < 0.05, **0.001 < *p* < 0.01, ***p < 0.001; ns. indicated no statistical difference.

## Data Availability

The original contributions presented in the study are included in the article/supplementary material, further inquiries can be directed to the corresponding author.
